# Combined presigmoid retrolabyrinthine and retrosigmoid approach for large vestibular schwannoma: a case report

**DOI:** 10.3389/fsurg.2026.1817241

**Published:** 2026-05-11

**Authors:** Mitsuru Kojima, Ryota Tamura, Konosuke Ishikawa, Taichi Sayanagi, Kosuke Karatsu, Ryo Ueda, Masahiro Toda

**Affiliations:** Department of Neurosurgery, Keio University School of Medicine, Tokyo, Japan

**Keywords:** combined approach, presigmoid approach, retrolabyrinthine approach, retrosigmoid approach, vestibular schwannoma

## Abstract

**Background:**

The retrosigmoid approach is widely used for vestibular schwannoma resection. If the tumor is large, significant cerebellar retraction may be required, which can cause cerebellar edema. The presigmoid retrolabyrinthine route preserves the labyrinth and reduces cerebellar retraction and manipulation but provides only a narrow surgical corridor. We report the use of a combined approach to safely resect a large vestibular schwannoma with brainstem compression while avoiding complete postoperative deafness.

**Methods:**

A 24-year-old man presented with a large right vestibular schwannoma causing considerable brainstem compression. A combined temporal–suboccipital craniotomy with near-total mastoidectomy was performed to expose the sigmoid sinus while preserving the labyrinth. Microsurgical dissection was performed via the retrosigmoid corridor, while a rigid endoscope inserted through the presigmoid route provided direct visualization of the brainstem side.

**Results:**

This dual-corridor, dual-visualization technique enabled safe subperineural tumor removal and identification of the facial nerve. Gross total resection was achieved, and the postoperative course was uneventful. At approximately 6 months postoperatively, facial nerve function remained normal (House–Brackmann grade I). Formal postoperative pure-tone audiometry demonstrated residual hearing on the operated side, predominantly in the low-frequency range. Although serviceable hearing was not preserved, complete postoperative deafness was avoided.

**Conclusion:**

In large vestibular schwannomas with vertical brainstem extension, retrosigmoid resection alone may require excessive cerebellar retraction. Adding a presigmoid retrolabyrinthine corridor improves brainstem access, minimizes cerebellar retraction and manipulation, and facilitates safe tumor removal. This combined approach is a practical strategy for patients with significant brainstem compression.

## Introduction

The retrosigmoid (lateral suboccipital) approach is a versatile and commonly employed route to the cerebellopontine angle (CPA). It is widely used for resection of vestibular schwannomas owing to its wide operative field and direct visualization of neurovascular structures (1, 2). However, cerebellar retraction is required. Excessive retraction may result in venous congestion and cerebellar edema; moreover, it may cause hearing impairment related to traction injury of the cochlear nerve. The retrolabyrinthine approach, which minimizes cerebellar retraction and manipulation, is an alternative route to access the CPA. Since this approach preserves the semicircular canals, exposure of the internal auditory canal (IAC) can be technically challenging. In addition, the working space around the IAC that it provides tends to be narrow.

We report a patient with a large vestibular schwannoma causing significant brainstem compression in whom both retrosigmoid and presigmoid (retrolabyrinthine) corridors were used to resect the tumor. A surgical microscope was employed through the retrosigmoid space and an endoscope through the presigmoid corridor. Gross total resection was achieved, facial nerve function remained House–Brackmann grade I, and postoperative audiometry confirmed residual hearing on the operated side, thereby avoiding complete deafness.

## Case description

A 24-year-old man with a 1-year history of gradual right-sided hearing disturbance and strabismus underwent magnetic resonance imaging (MRI) of the brain, which showed a right vestibular schwannoma, prompting referral to our department. On examination, the patient's Glasgow coma scale score was 15. Pure-tone audiometry revealed right-sided sensorineural hearing loss with a four-frequency pure-tone average of 46.3 dB. Speech audiometry showed 75% discrimination at 60 dB, corresponding to Gardner–Robertson hearing grade II. Auditory brainstem responses showed no identifiable wave V. Additionally, mild sensory disturbance was noted in the maxillary division of the right trigeminal nerve. Facial nerve function was normal (House–Brackmann grade I).

**Figure 1:**
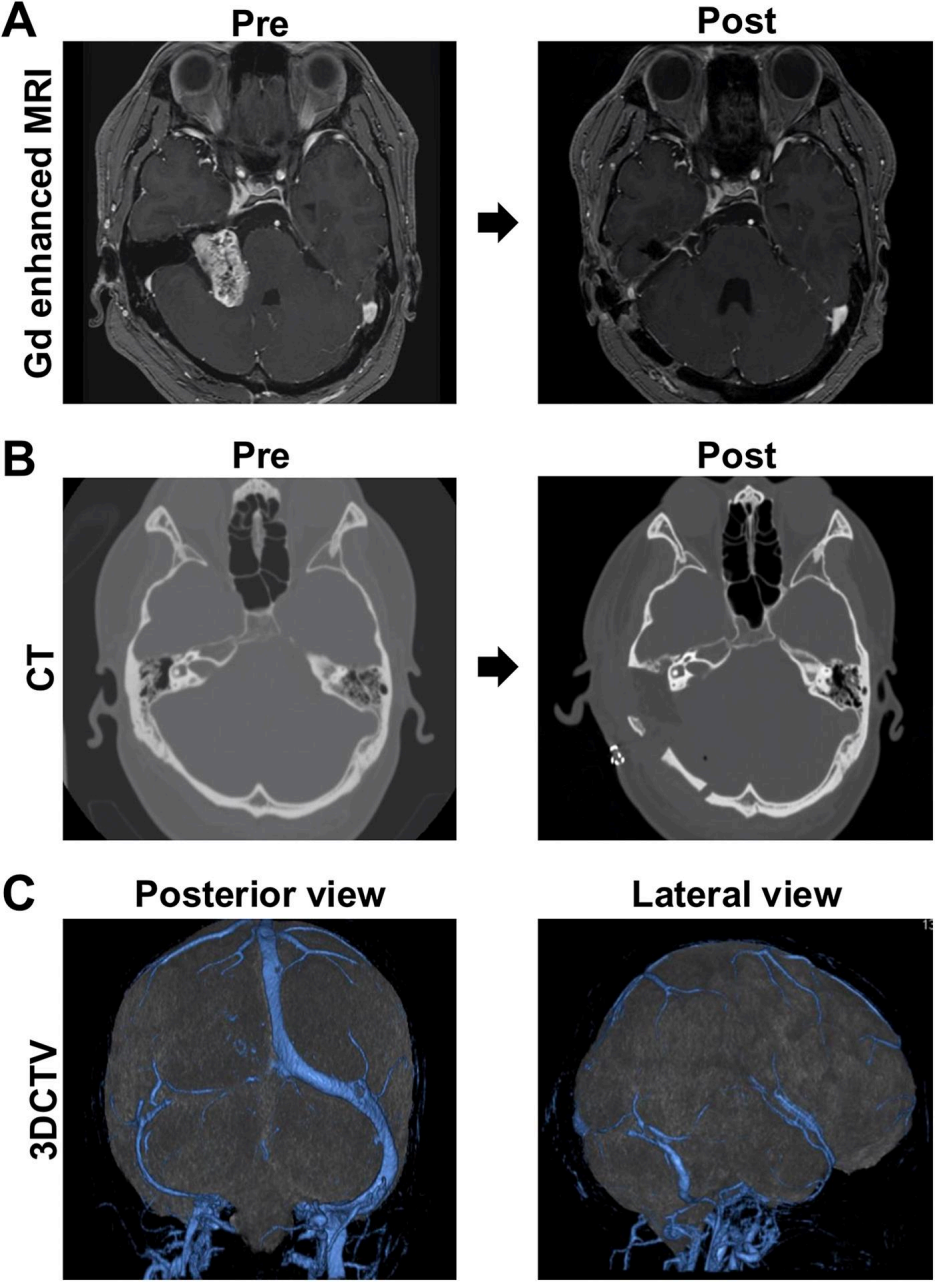
Preoperative and postoperative radiographic findings. MRI demonstrates a 40 mm × 27 mm × 29 mm tumor showing heterogeneous enhancement, extending vertically toward the brainstem and cerebellar peduncle along the surface of the petrous bone, with significant compression of the brainstem. Gross total resection was maintained without evidence of recurrence on MRI at approximately 6 months postoperatively **(A)**. Postoperatively, the internal auditory canal was opened, and mastoidectomy was performed up to the area immediately adjacent to the semicircular canals **(B)**. Preoperative three-dimensional CT venography, viewed from an oblique posterior angle, demonstrates that the ipsilateral sigmoid sinus is present but smaller in caliber than the contralateral side **(C)**.

MRI demonstrated an approximately 40 × 27 × 29 mm heterogeneously enhancing tumor expanding the IAC, which remained patent at the fundus (Figure 1A). The tumor markedly compressed the brainstem dorsally, while the sixth and lower cranial nerves were spared. It extended vertically along the petrous surface toward the cerebellar peduncle, corresponding to Koos grade IV. Tractography and Fast Imaging Employing Steady-state Acquisition imaging after contrast administration suggested that the facial nerve coursed anteromedially along the ventral surface of the tumor.

Preoperative three-dimensional CT venography, including an oblique posterior view, demonstrated that the ipsilateral sigmoid sinus was present but smaller in caliber than the contralateral side. This venous anatomy was taken into consideration during planning of the combined presigmoid retrolabyrinthine and retrosigmoid approach (Figure 1C).

## Surgical procedures

**Figure 2:**
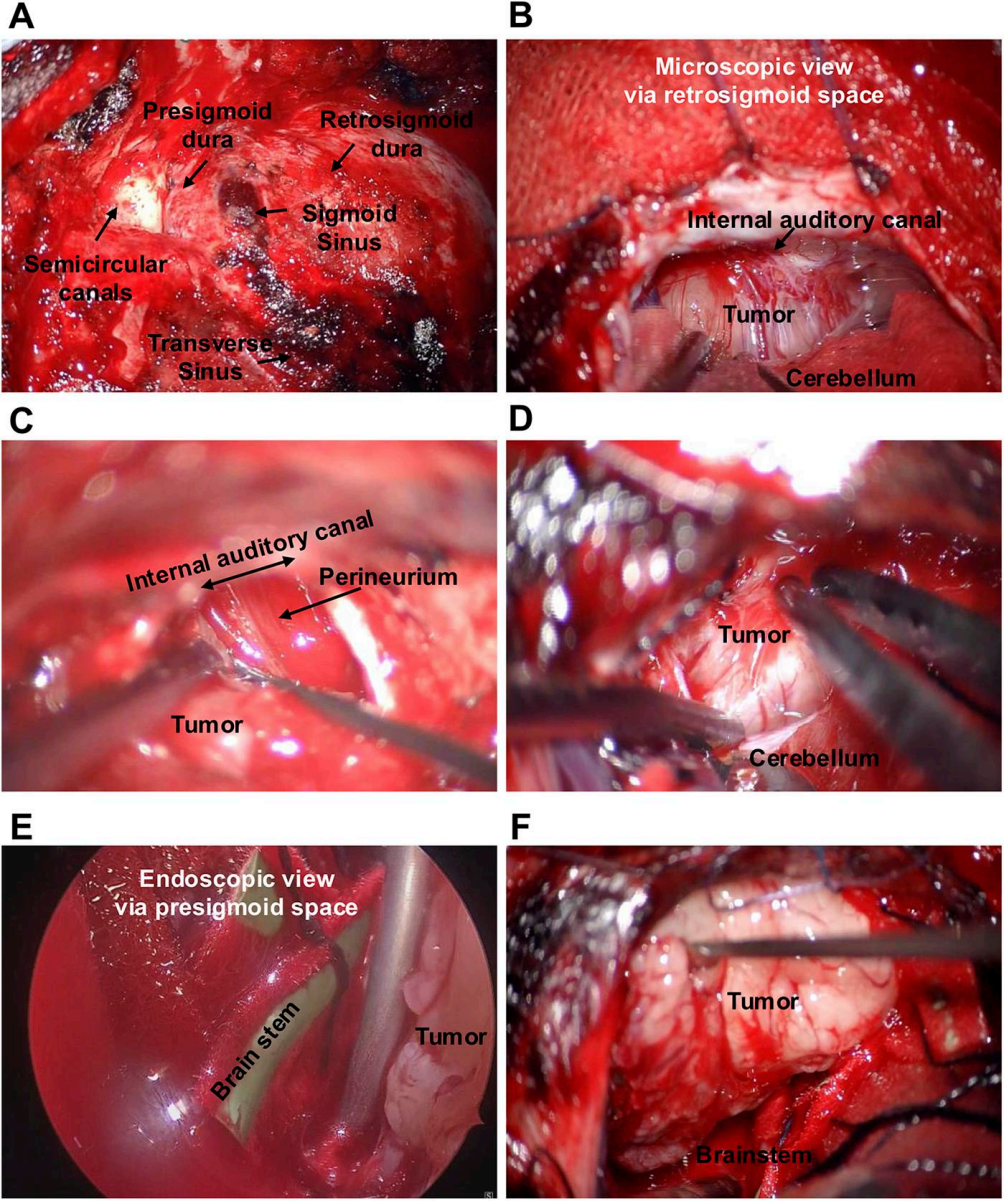
Intraoperative findings. Lateral suboccipital craniotomy and mastoidectomy were performed up to the area immediately adjacent to the semicircular canals, exposing the transverse sinus, sigmoid sinus, and the retro- and presigmoid dura **(A)**. The tumor was identified under the operating microscope via retrosigmoid space **(B)**. The internal auditory canal was opened, and the intracanalicular portion of the tumor was dissected in a subperineural plane **(C)**. Subperineural dissection was also carried out from the brainstem side **(D)**. Endoscopic assistance was used for tumor removal around the brainstem via presigmoid space **(E)**. Finally, the tumor was removed completely **(F)**.

After induction of general anesthesia, the patient was placed in the park-bench position with the head fixed horizontally and slightly vertex-down. Four burr holes were placed around the transverse sinus, and a combined temporal and suboccipital craniotomy was performed. A near-total mastoidectomy was then carried out. A combined temporal-suboccipital craniotomy and mastoidectomy were planned preoperatively to allow opening of the presigmoid corridor if visualization of the tumor-brainstem interface proved inadequate through the retrosigmoid route alone. The cortical bone was removed, and the sigmoid sinus and digastric ridge were exposed. The mastoid antrum was opened around Henle's spine, and the lateral semicircular canal was identified. Bone between the canal and sigmoid sinus was removed, and the presigmoid dura was carefully dissected free. The endolymphatic sac was divided, and the dura was peeled down to the jugular bulb. The posterior and superior semicircular canals were skeletonized, and the fallopian canal was thinned under direct nerve stimulation to confirm the facial nerve (Figures 1B, 2A).

Microsurgical dissection then proceeded. Cerebrospinal fluid was released from the cisterna magna, and a dural incision was made along the sigmoid sinus, with the mastoid bone already drilled open, allowing the retrosigmoid space to be handled quite widely (Figure 2B). The tumor surface was stimulated directly, and no facial nerve response was elicited. Approximately 6 mm of the posterior wall of the IAC was drilled away, the IAC dura incised, and the tumor capsule opened. Subperineural dissection was carried out toward the fundus (Figure 2C), where direct stimulation evoked responses from the orbicularis oculi and oris muscles, confirming the facial nerve course anteromedially.

**Figure 3:**
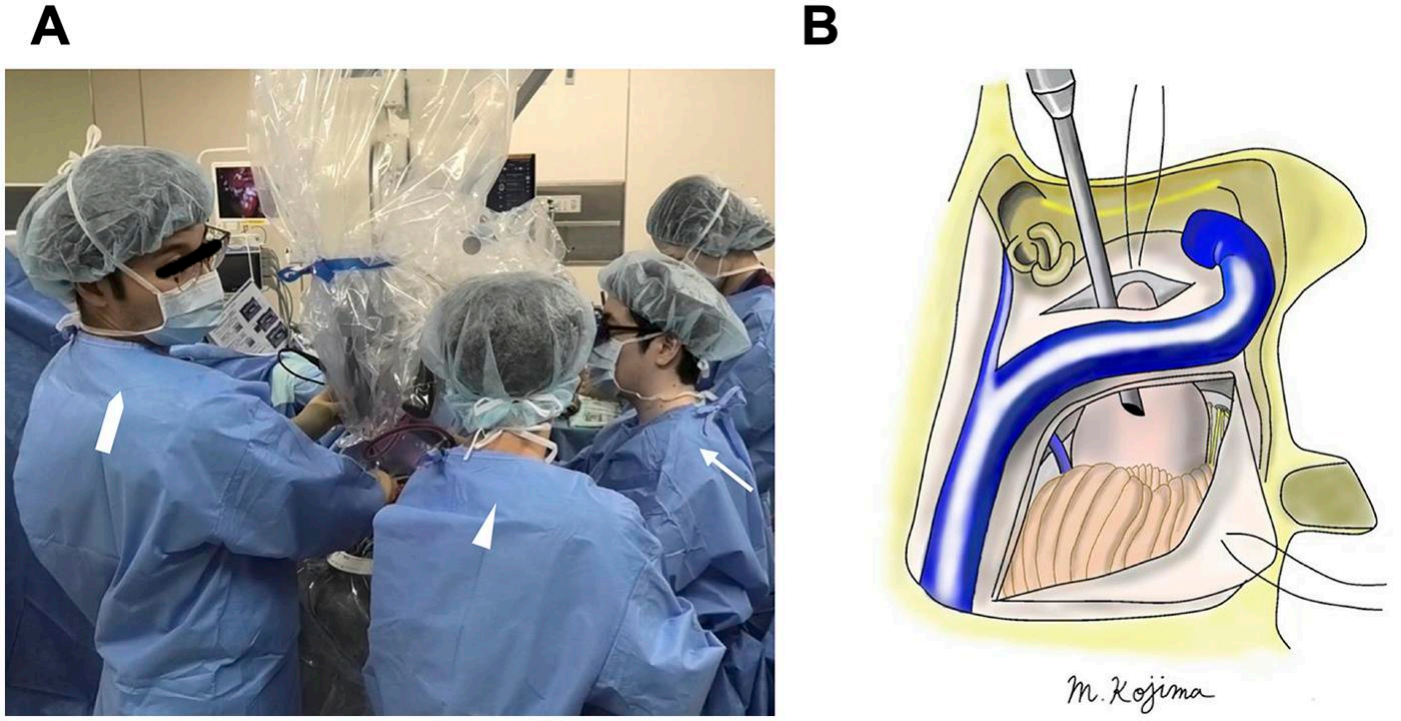
Operating surgeon and assistants. Intraoperative views showing the operating surgeon at the microscope [**(A)**, arrow], the microsurgical assistant [**(A)**, arrowhead], and the endoscopic assistant [**(A)**, thick arrow]. Schematic illustration demonstrates the overview of combined presigmoid retrolabyrinthine and retrosigmoid approach **(B)**.

Stepwise subperineural dissection proceeded from the IAC to the cisternal portion, preserving the perineurium around the facial nerve. As tumor debulking progressed, the facial nerve became visible and responded sharply to transcranial facial motor evoked potential stimulation. When proceeding toward the brainstem, it became evident that excessive cerebellar retraction would be required to adequately visualize the tumor and its surrounding structures; therefore, the presigmoid route was opened. The dura of the presigmoid space was incised, providing access to the brainstem without significant cerebellar retraction (Figure 2D). A rigid Storz endoscope was inserted through the presigmoid corridor to monitor brainstem and vascular structures (Figure 2E) while the microsurgical instruments were manipulated through the retrosigmoid window. This bimanual, dual-visualization technique allowed safe dissection without instrument interference (Figures 3A,B). The tumor was removed, and gross total resection was confirmed visually (Figure 2F). Fat harvested from the abdominal wall was packed into the mastoid cavity to obliterate the air cells. Dural augmentation was performed using autologous fascia, and the bone flap was replaced.

## Results

Computed tomography (CT) performed immediately after surgery demonstrated no evidence of hemorrhage or hydrocephalus. The patient's motor function was intact, and facial nerve function remained normal (House–Brackmann grade I). Mild right facial numbness persisted postoperatively.

Early mobilization was initiated on postoperative day 1. Magnetic resonance imaging (MRI) obtained on postoperative day 5 confirmed gross total tumor resection. Transient postoperative vertigo and nausea gradually improved, and the patient was discharged home on postoperative day 9 with a modified Rankin Scale score of 0.

At approximately 6 months after surgery, facial nerve function remained normal (House–Brackmann grade I). Formal postoperative pure-tone audiometry demonstrated residual hearing on the operated side, predominantly in the low-frequency range, with a four-frequency pure-tone average of 93.8 dB. Postoperative speech discrimination testing was not available. Although serviceable hearing was not preserved, complete postoperative deafness was avoided. Follow-up MRI at 6 months demonstrated maintained patency of the sigmoid sinus without evidence of stenosis or thrombosis. No cerebrospinal fluid leakage was observed during the postoperative course or at approximately 6 months of follow-up.

## Discussion

In this case, the tumor extended vertically against the petrous surface and severely compressed the brainstem, rather than spreading along the petrosal plane. The standard approaches for vestibular schwannoma include the retrosigmoid, translabyrinthine, retrolabyrinthine, and middle fossa routes (1). Among these, the retrosigmoid approach remains the most popular and versatile (2), but significant cerebellar traction required to expose large tumors can cause edema and venous congestion, compromising the surgical corridor; moreover, it may result in hearing impairment caused by excessive traction on the cochlear nerve. The translabyrinthine approach, which involves both mastoidectomy and labyrinthectomy, offers a straight route to the IAC and CPA, but sacrifices hearing (3). The retrolabyrinthine approach, in contrast, preserves the semicircular canals and potentially maintains hearing, but the working space is relatively restricted (4). Reports have shown that cerebellar edema and postoperative rehabilitation needs are more frequent with the retrosigmoid approach than the translabyrinthine (5).

Given the tumor's vertical growth and brainstem compression, we anticipated that excessive cerebellar retraction would be required if a solely retrosigmoid approach was employed. Therefore, to improve access to the brainstem side while preserving the possibility of residual postoperative hearing, we elected to perform a combined presigmoid retrolabyrinthine and retrosigmoid approach.

Kleijwegt et al. described a “360° around the sigmoid sinus” combined retrosigmoid–translabyrinthine approach that preserves the sinus while allowing dual-sided access for giant CPA tumors (6). Although effective, this method is technically demanding and associated with risks of cerebrospinal fluid leakage, venous injury, and complete hearing loss. In 1995, Silverstein et al. reported the combined retrolabyrinthine and retrosigmoid approach; here, the retrolabyrinthine approach is added to avoid the cerebellar retraction-associated complications inherent to the retrosigmoid approach. With the combined retrolabyrinthine and retrosigmoid approach, unlike the previously mentioned 360° exposure, a limited mastoidectomy is performed. The sigmoid sinus is exposed along its entire length, and the dural incision is placed parallel to the posterior 3 mm of the sinus. The sinus is gently retracted anteriorly using stay sutures, thereby minimizing cerebellar traction (7).

Our procedure involved a near-total mastoidectomy with full 360° exposure of the sigmoid sinus while preserving the labyrinthine capsule and fallopian canal—representing an intermediate strategy between those two methods. An endoscope was introduced through Trautman's triangle via the presigmoid space to visualize the brainstem side while microsurgical manipulation proceeded through the retrosigmoid window. The magnified view of the endoscope facilitated clear identification of the perineurial layer, enabling safe subperineural dissection.

Opening the sigmoid sinus circumferentially allowed a 360° working angle, providing a line of sight comparable to that achieved via a presigmoid approach, even from the retrosigmoid corridor. Several authors have reported endoscope-assisted (8–10) or purely endoscopic (11, 12) vestibular schwannoma resections via the presigmoid or retrosigmoid routes, particularly in patients with well-pneumatized temporal bones, enabling tumor removal with minimal cerebellar retraction (8–10). However, these approaches remain technically demanding because of the narrow operative corridor and inevitable instrument interference (10).

A known risk of presigmoid/posterior petrosal approaches is postoperative sigmoid sinus stenosis or thrombosis caused by manipulation or compression during sinus skeletonization (13). The reported incidence ranges from approximately 4.7% to 11.6%. It is usually asymptomatic but occasionally leads to venous infarction, cerebellar edema, or dural arteriovenous fistula formation (13–15). The delayed mechanism is thought to involve postoperative edema or external compression causing endothelial injury and secondary thrombosis (16). In our patient, the ipsilateral sigmoid sinus was present, although smaller in caliber than the contralateral side on preoperative venous imaging. This anatomical feature may have contributed to the technical feasibility of the present approach. Despite extensive sigmoid sinus skeletonization and manipulation, follow-up MRI at 6 months demonstrated preserved sinus patency without evidence of stenosis or thrombosis. However, whether this approach can be applied with the same safety and utility in patients with a more developed sigmoid sinus remains uncertain. Although it may still be feasible in such patients, further investigation is necessary to clarify its indications, safety, and reproducibility.

This report describes a single case, and the clinical outcomes cannot be generalized. Although postoperative audiometry was eventually obtained, the hearing outcome should be interpreted with caution and should not be overstated as “hearing preservation” in the strict functional sense. Formal audiometric evaluation demonstrated that serviceable hearing was not maintained, despite the presence of limited residual hearing, predominantly in the low-frequency range. Therefore, we believe that the most appropriate interpretation of this case is not that functional hearing was preserved, but rather that this dual-corridor strategy enabled gross total resection and preservation of House–Brackmann grade I facial nerve function while avoiding complete postoperative deafness in a highly selected patient with favorable venous anatomy.

## Conclusion

For large vestibular schwannomas extending vertically to the brainstem, the conventional retrosigmoid approach alone may entail excessive cerebellar retraction. By adding a mastoidectomy and utilizing the presigmoid corridor under endoscopic guidance, we were able to perform delicate microsurgical dissection from the retrosigmoid side while minimizing cerebellar compression. Although postoperative audiometry was eventually obtained, the hearing outcome should be interpreted with caution and should not be overstated as “hearing preservation” in the strict functional sense. Formal audiometric evaluation demonstrated that serviceable hearing was not maintained, despite the presence of limited residual hearing, predominantly in the low-frequency range. Therefore, we believe that the most appropriate interpretation of this case is not that functional hearing was preserved, but rather that this dual-corridor strategy enabled gross total resection and preservation of House–Brackmann grade I facial nerve function while avoiding complete postoperative deafness in a highly selected patient with favorable venous anatomy.

## Data Availability

The original contributions presented in the study are included in the article/Supplementary Material, further inquiries can be directed to the corresponding author.
