# Supplementary material for: Combined presigmoid retrolabyrinthine and retrosigmoid approach for large vestibular schwannoma: a case report
Source: Front Surg. 2026 May 11;13:1817241. doi: 10.3389/fsurg.2026.1817241 (PMC13199334; doi:10.3389/fsurg.2026.1817241)
Supplement: Supplementary file 2 [file Supplementaryfile1.docx]

Supplementary Material

**Supplementary Video**

A combined temporal and suboccipital craniotomy with mastoidectomy was performed to fully expose the transverse sinus and sigmoid sinus, thereby establishing both retrosigmoid and presigmoid operative corridors. Microsurgical dissection was initiated via the retrosigmoid approach. After gentle cerebellar retraction, the duplicated arachnoid membrane was sharply dissected to access the tumor. Careful inspection confirmed the absence of facial nerve fibers on the tumor surface, after which the tumor capsule was incised and internal decompression was performed.

The posterior wall of the internal auditory canal was then unroofed, and the IAC dura was opened to expose the intracanalicular component of the tumor. The distal portion of the intracanalicular tumor was secured, and dissection was advanced toward the cisternal portion with preservation of the perineurium. The cisternal component was subsequently dissected in a subperineural plane in both cranial and caudal directions and connected to the component dissected from within the internal auditory canal.

Finally, the tumor was completely removed from the brainstem side with continued  preservation of the perineurium, resulting in gross total removal.

The video demonstrates the simultaneous use of microscopic and endoscopic techniques. A rigid endoscope was introduced through the presigmoid corridor to continuously monitor the cerebellum, brainstem, and adjacent vascular structures, ensuring the absence of excessive tension, while microsurgical manipulation was performed through the retrosigmoid corridor. This dual-corridor, dual-visualization strategy enabled safe tumor removal with preservation of the perineurium and without instrument interference.
